# Veterinary Herd Health Consultancy and Antimicrobial Use in Dairy Herds

**DOI:** 10.3389/fvets.2020.547975

**Published:** 2021-02-02

**Authors:** Nanna K. Skjølstrup, Liza R. Nielsen, Carsten S. Jensen, Dorte B. Lastein

**Affiliations:** ^1^Section for Production, Nutrition and Health, Department of Veterinary and Animal Sciences, Faculty of Health and Medical Sciences, University of Copenhagen, Frederiksberg, Denmark; ^2^Section for Animal Welfare and Disease Control, Department of Veterinary and Animal Sciences, Faculty of Health and Medical Sciences, University of Copenhagen, Frederiksberg, Denmark; ^3^Department of Sociology, Faculty of Social Sciences, University of Copenhagen, Copenhagen, Denmark

**Keywords:** antimicrobial use, antimicrobial resistance, veterinarians, farmers, veterinary herd health consultancy, decision-making, social factors, dairy cattle

## Abstract

The globally increasing level of antimicrobial resistance affects both human and animal health, why it is necessary to identify ways to change our current use of antimicrobials. The veterinary herd health collaboration between veterinarians and dairy farmers provides a useful setting for changing antimicrobial use in livestock. However, farmers and veterinarians work in a complex agricultural setting influenced by socio-economic factors, which complicates their choices regarding antimicrobial usage. It is therefore necessary to be aware of the range of potential influencing factors and to integrate this knowledge in the relevant local settings. This manuscript presents a literature review of relevant factors relating to antimicrobial use within the veterinary herd health consultancy setting, including knowledge gaps of relevance for changing the use of antimicrobials. An enriched version of the framework of the Theory of Planned Behaviour was used to organise the literature review. We identified diverging attitudes on correct treatment practices and perceptions of antimicrobial resistance among veterinarians and farmers, influenced by individual risk perception as well as social norms. Furthermore, disagreements in terms of goal setting and in the frequency of herd visits in relation to herd health consultancy can negatively influence the collaboration and the intention to change antimicrobial use. Farmers and veterinarians emphasise the importance of legislation and the role of the dairy industry in changing antimicrobial use, but the relevance of specific factors depends on the country-specific context. Overall, farmers and veterinarians must communicate better to understand each other's perspectives and establish common goals within the collaboration if they are to work efficiently to reduce antimicrobial use. Farmers and veterinarians both requested changes in individual behaviour; however, they also called for national and structural solutions in terms of balanced legislation and the availability of better diagnostics to facilitate a change in antimicrobial use practices. These various paths to achieving the desired changes in antimicrobial use illustrate the need to bridge methodological research approaches of veterinary science and social sciences for a better understanding of our potential to change antimicrobial use within the dairy farm animal sector.

## Introduction

Antimicrobial use (AMU) is important to consider as antimicrobial resistance (AMR) is increasing globally, affecting both human, and animal health (1, 2). Within the farm animal sector, veterinarians are responsible for the use of antimicrobials in collaboration with the farmer. This specific interaction should therefore be taken into account when promoting “rational AMU,” which here is defined as a limitation in inappropriate use, as well as a reduction in the need for antimicrobials. This definition has been adapted from the European Commission, which characterises inappropriate use as “use in an untargeted manner, at sub-therapeutic doses, repeatedly, or for inappropriate periods of time” (3). With this in mind, the veterinary herd health consultancy (VHHC), which frames the collaborative work between the veterinarian and the farmer at dairy farms around the world, comprises an interesting study case with regard to promoting rational AMU. The majority of antimicrobials currently used in dairy cattle are used to treat and control mastitis [(4), p. 22–3] and pneumonia in calves [(5), p. 5–6], and these diseases are therefore central topics in the work on rational AMU.

Over time, veterinary tasks in dairy herds have changed character. Previously, the focus was primarily on the treatment and prescription of medicine, but there has more recently been a shift towards disease prevention [(6, 7), p. 11–6]. With the introduction of epidemiology into veterinary science, the collection and analysis of quantitative data in veterinary practices has led to an acknowledgement that production diseases are multifactorial and connected with housing, nutrition, genetics, and other diseases. The concept of herd health management (HHM) was introduced and characterised as “an integrated, holistic, proactive, data-based, and economically framed approach to prevention of disease and enhancement of performance” by LeBlanc et al. [(8), p. 1267]. The HHM approach and research within the area have inspired practitioners globally to introduce, advise on and apply preventive measures related to herd-level health and production, often through data- and knowledge-driven engagement on farms (9–11). The HHM approach ideally implies a continuous collaboration between the farmer and the veterinarian, as the same veterinarian will often be affiliated with a farm over long periods of time. This close collaboration, in combination with a focus on herd health and production, provides a suitable setting for working explicitly towards rational AMU. The specific HHM approach differs from country to country (6, 10, 12, 13), but the type of VHHC in focus in this review article is defined by a continuous collaboration between a farmer and the same veterinarian, with regular herd visits and a focus on herd health and production.

Research shows that the traditional focus on quantitative data analysis and economics embedded within the HHM approach does not motivate all farmers to change their behaviour (14–17), and factors relating to farmers' and veterinarians' decision-making processes in particular need further investigation (18, 19). Farmers and veterinarians act in a complex agricultural context characterised by legislation on AMU, changing incomes due to fluctuating milk prices, the physical condition of the farm, farm and veterinary businesses aiming to make a profit, and social norms to which farmers and veterinarians try to adhere. All of these factors could potentially affect the choice to use antimicrobials rationally, implying the need to understand and take such “qualitative” factors into consideration as a part of the VHHC when working to change behaviour.

We argue that the choices made by farmers and/or veterinarians either individually or in collaboration, for example whether or not to prescribe or treat an animal, are the starting point for working towards rational AMU in dairy cattle. Our focus is therefore on the factors that influence behaviour in terms of rational AMU within farmers' and veterinarians' collaborative framework. Possible influencing factors affecting individual and/or collaborative AMU choices must be identified and considered from an overall sociological perspective. The VHHC could then not only be expanded to include quantitative data on health, production and economics as part of a motivation for change but it could also be broadened to take farmer- and veterinarian-specific motivational factors into consideration.

However, not all factors are equally important to every farmer and veterinarian. Each farmer is a unique individual, and a personal approach should be taken within a specific-herd context [(20), p. 3330, (16), p. 13]. Furthermore, the local agricultural setting differs from country to country, which is why identified factors might not all be of equal importance across all countries. It is therefore necessary to relate every identified factor to the country of interest, with its national context-specific barriers and opportunities [(21), p. 160–1].

The overall objective of this study was to improve the understanding of relevant factors for achieving rational AMU within the collaborative context of VHHC in dairy cattle herds. The first sub-objective was to review, summarise and discuss the factors of relevance for VHHC and rational AMU in dairy herds. Furthermore, the findings are discussed from a socio-economic perspective to broaden the understanding of their meaning. The second sub-objective was to identify knowledge gaps of relevance for changing AMU practices within the VHHC setting, as well as challenges and opportunities for future research.

The initial inclusion criterion for the literature search was studies on dairy cattle and AMU (other types of medication were excluded). Secondly, studies had to be conducted in an intensive production context characterised by high milk production and relation to a global market, as we argue that there are different AMU-related factors at play in intensive and extensive farming. Finally, studies had to place an emphasis on the farmer–veterinarian relationship. The review began with a systematic literature search across seven databases, and 39 out of 122 articles were used in this review after screening for relevance. We also included additional articles conducted within the social science research field to discuss and elaborate on the multifaceted area of AMR research.

## Overview of Factors Affecting the Intention to Move Towards Rational Antimicrobial use: A Socio-Ecological Model

Numerous factors influencing the intention to change AMU were identified in the literature search. We used a model developed and previously used in the disease prevention and control context as a structural framework to organise these factors and to give a better overview [(22), p. 278]. Their model was originally built upon a study by Panter-Brick et al. [(23), p. 2813] and was developed to illustrate barriers to the control of zoonotic diseases. Controlling zoonotic diseases is complex and unpredictable aspects must be taken into consideration, such as future consequences that can be difficult to comprehend and react on in the present, as is the case with AMR. The model was chosen due to its more holistic approach, taking into account the agricultural setting.

The model proposes that a person's intention to perform a certain behaviour can be explained by three factors: (1) the person's attitude, (2) the person's subjective norm and (3) the person's perceived behavioural control [(24), p. 179–82]. The original model builds on the Theory of Planned Behaviour, but has been further developed here in order to take extrinsic (e.g., national, regional, and herd-specific) factors more directly into account [(23), p. 2811–2]. Panter-Brick et al. (23) argued that intention to change is not driven solely by intrinsic factors, making the model socio-ecological and combining social and physical aspects of the individual. In our case, the relationship between two groups of individuals (farmers and veterinarians) and their cooperation within VHHC in the agricultural context is important. Therefore, we further developed the model to contain and organise the intrinsic and extrinsic factors while allowing for the factors to be either specific to one group or common to both.

The original Theory of Planned Behaviour and its focus on individual behaviour has been criticised on a number of occasions, especially in relation to its behaviourist foundation [(25), p. 5–10, (26), p. 3, (27), p. 1–3]. In this context, we do not use the model as an argument for behaviourism but as a framework for structuring the research of different empirical aspects and contexts where the farmer–veterinarian interaction can influence the use of antimicrobials. The model helps us present an overview of areas and situations that influence AMU, both at individual and farm level, but also from a broader societal perspective. This is in line with research within the social sciences, where the structural dimensions related to AMU (e.g., social, economic, and biological factors) are investigated [(25), p. 8–10; (28), p. 2].

The intrinsic factors in the model consist of three groups: (1) Behavioural beliefs representing a person's attitudes, which are often defined by core values; (2) Normative beliefs defined by social norms and how the individual perceives these; (3) Belief in self-efficacy, which is closely related to a person's trust in their own ability to carry out the change.

The extrinsic factors also consist of three groups: (1) Community & Industry, including influence from the agricultural industry, and trade partners as well as the rural community; (2) Culture & Society, including national legislation and guidelines as well as influence from consumers; (3) Knowledge, Skills & Ability, relating to the overall availability of important resources, finances, knowledge, and tools for possible change (22).

**Figure 1:**
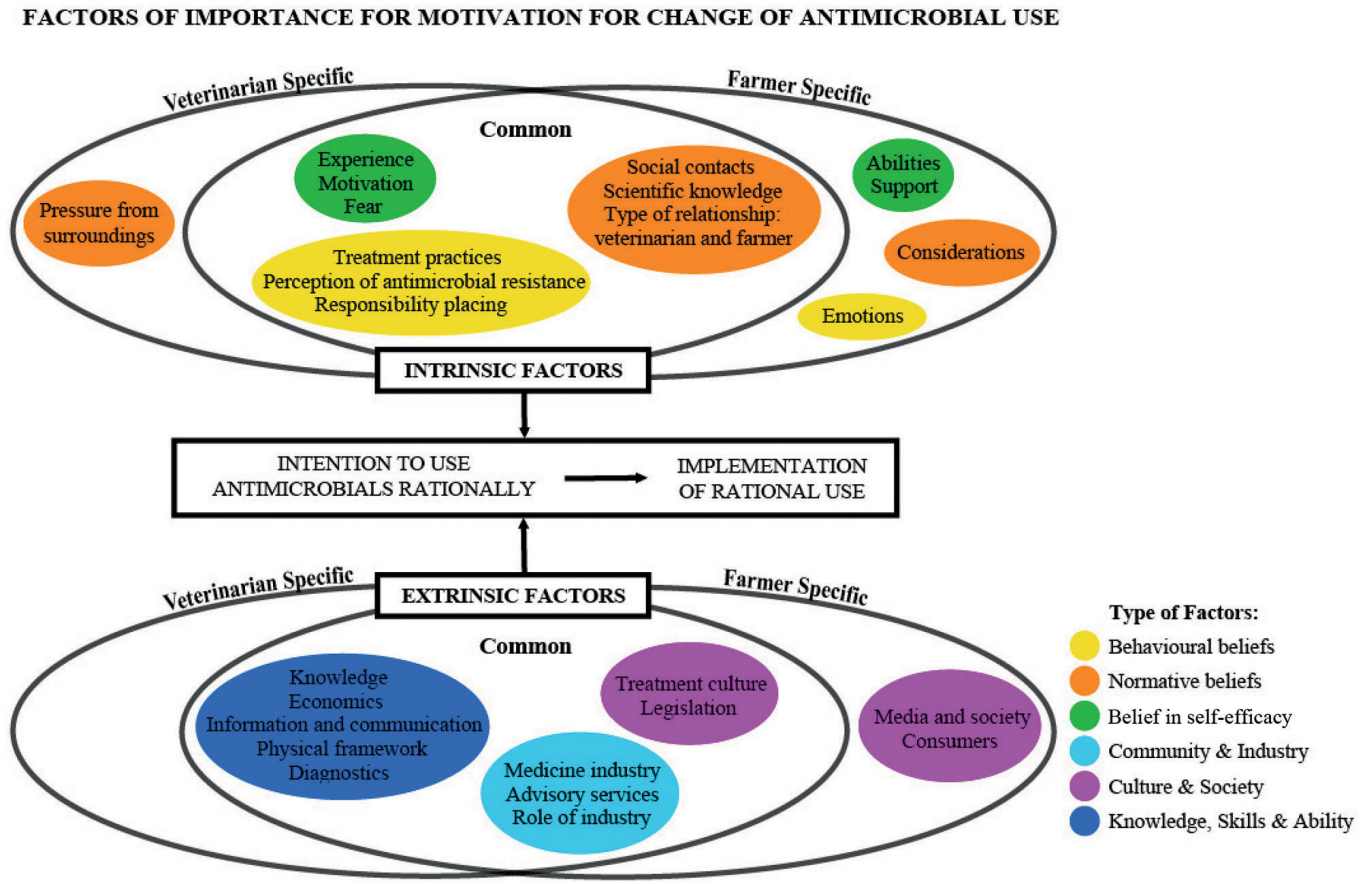
Factors influencing AMU. Some factors have been identified as common to both veterinarians and farmers, whereas others are only identified for the one or the other. The model was inspired by Ellis-Iversen et al. (22).

Figure 1 shows how the findings from the reviewed papers have been embedded in the model. The identified factors appear as headlines placed within the appropriate section of the model, e.g., concerning either one of the groups within the intrinsic or extrinsic factors, and relevant to either the farmer, the veterinarian or both. For each headline, e.g., “Responsibility placing,” several studies may have contributed findings to support the importance of this factor. In the next section, the identified factors and the related literature are presented according to the structure of the model, starting with the intrinsic factors and ending with the extrinsic factors. It is important to note that only factors that were identified in the literature are summarised and discussed.

## Intrinsic Factors Affecting the Intention to Move Towards Rational Antimicrobial Use

Research on intrinsic factors related to an individual's attitudes, core values, perception of social norms and belief in their own ability to change will be presented in the following sections.

### Differences in Veterinarian and Farmer Attitudes on Antimicrobial Use

The first of the intrinsic factors are behavioural belief-based factors, concerning personal attitudes. The first point to be presented here, “Treatment practices” (Figure 1, intrinsic, common factor), appeared to be an important point for both veterinarians and farmers, with a range of attitudes on how to approach treatment. Several studies suggest that veterinarians and farmers both agree that sick animals need treatment [(29), 44–5, (30), p. 86, (31), p. 4, (32), p. 7], yet motives seem to differ between the two groups. According to Speksnijder et al. [(29), p. 44], veterinarians regard diseased animals from a professional and ethical point of view, with treatment primarily related to their perceived obligation as a veterinarian to ensure animal health and welfare. For farmers, treatment appears to be driven by a focus on animal welfare and an urge to stop individual animal suffering [(33), p. 112–3]. However, the threshold for treatment can change, e.g., alleviating suffering among diseased animals can be addressed by intense follow-up instead of immediate treatment [(34), p. 1845–7]. Both studies identified practical farmer-specific issues related to having sick animals: it is time-consuming and interrupts the daily routine, and it can be economically challenging [(33), p. 114, (34), p. 1845–8]. Having sick animals is therefore a complex issue for both farmers and veterinarians, but it complicates different aspects for the two groups. For veterinarians, it is mostly about ethical and professional standards, whereas for farmers, the challenges are primarily related to practical issues and emotional frustration.

Other perceptions about “Treatment practices” have also been identified in the literature (Figure 1, intrinsic, common factor). Several studies found that both veterinarians and farmers believe that vaccination plays a key role in reducing AMU [(32), p. 11, (31), p. 7, (35), p. 5–7, (36), p. 3232–7]. However, vaccination was perceived as ineffective among farmers in Washington State [(36), p. 3234]. Differences in prescribing behaviour and AMU patterns have also been described. Misuse and illegal over-the-border purchases of antimicrobials have been identified among farmers [(36), p. 3233–4, (37), p. 56, (38), p. 3496, (39), p. 9–10], and veterinarians from France, the UK and Switzerland expressed their frustration with veterinary prescribing behaviour, either directed at themselves or their colleagues [(40), p. 67, (32), p. 9–12, (39), p. 8–10]. Alternative treatment methods were perceived by farmers as being too time-consuming [(33), p. 114–6, (34), p. 1845], and a distrust in their effect was also identified [(33), p. 115]. The attitudes behind or reasons for some of these treatment practices cannot always be elaborated upon due to the study design that is traditionally used within veterinary research, e.g., surveys and questionnaires. For example, the position on vaccination among farmers in Washington State was identified based on a questionnaire study (36), and this type of study rarely provides the reason behind an answer, as would be possible in an interview, for instance.

Looking into the social science literature from human medicine might provide some possible explanations for the identified treatment practices among veterinarians and farmers. For example, Broom et al. [(41), p. 1995] found a ritualised AMU among doctors. Specific treatments repeatedly resulting in positive outcomes might lead to both farmers and veterinarians believing that this treatment is more effective and therefore preferred. For the farmer, this might result in a request for specific antimicrobials, which he or she perceives as most effective. This could cause potential conflicts between veterinarians and farmers due to disagreements over drug preferences. Similarly, veterinary drug choices may be ritualised.

Responsibility for the rational use of antimicrobials and where this responsibility should be placed has also been studied (Figure 1, intrinsic, common factor, “Responsibility placing”). There is evidence in the literature that both farmers and veterinarians might perform “other-blaming” behaviour when placing responsibility for the rational use of antimicrobials. “Other-blaming” can be understood as viewing other people as responsible for causing an issue, while the individual's own behaviour is perceived as unproblematic. One study from the UK found that frustrations among farmers and veterinarians due to the physical framework of the livestock industry as well as a lack of stewardship among doctors contributed to other-blaming [(32), p. 7–12]. The same finding was reported in the Netherlands, where the misuse of antimicrobials by doctors and international traffic were seen as the primary causes of AMR by some interviewed veterinarians [(29), p. 45]. Dairy farmers interviewed in Tennessee believed that there was no connection between AMU in agriculture and public health risks. Instead, this risk was perceived as being linked to AMU in the human sector [(31), p. 7]. Renunciation of responsibility by both veterinarians and farmers can therefore act as a barrier to changing AMU.

In connection with this attitude and “Perception of AMR” (Figure 1, intrinsic, common factor), some literature suggests that farmers and veterinarians perceive their own AMU as an insignificant contributor to global AMR [(30), p. 87, (29), p. 44–5]. However, the opposite opinion was also identified [(42), p. 4, (29), p. 44, (36), p. 3235], as well as an experience-dependant factor as more experienced veterinarians seem to be less aware of the potential risks related to antimicrobial overuse [(43), p. 367]. A survey completed by veterinarians from New Zealand found that younger veterinarians were more likely than older ones to perceive AMR as a risk [(30), p. 88]. An ethnographic study conducted at a dairy farm in the UK concluded that the perception of AMR as a risk is related to knowledge. The study argues that knowledge of AMR within agriculture is based to a large extent on practical experiences in specific farm contexts. Due to microbial culturing not being used at farm level, other factors will often outweigh resistance as plausible explanations for treatment failure [(44), p. 1–9]. To elaborate on the risk perception of AMR experienced by farmers and veterinarians, inspiration can be found in research conducted within human medicine. Doctors must balance the acute risk of losing a patient in need of antimicrobial treatment and the global, long-term risk of AMR [(45), p. 828–30]. Similarly, this could be an underlying mechanism explaining why farmers and veterinarians do not see their own AMU as a significant contributor to AMR globally; perhaps the acute risk of losing an animal takes priority over the long-term perspective of AMR, thus “forcing” the farmer or veterinarian to ignore the risk of resistance. In a similar way to the renunciation of responsibility mentioned earlier, the perception of own AMU as an insignificant contributor to global AMR can lower the intention to change AMU [(44), p. 1–9].

Literature suggests that “Emotions” can shape farmers' attitudes (Figure 1, intrinsic, farmer factor). Fischer et al. [(46), p. 2729] found that farmers felt frustrated when they had sick animals, and Vaarst et al. [(33), p. 113] found that “favourite cows” could receive treatment even when the prognosis was poor. This point about emotions was not found for veterinarians in the literature. Research on the prescribing behaviour of doctors identified the need to “just do something” when patients were close to dying [(41), p. 1999]. This feeling of at least trying to do something might be similar to the one experienced by farmers—and is potentially also evident for veterinarians.

As illustrated by the behavioural belief-based factors identified in the literature, farmers and veterinarians have different opinions regarding AMU and AMR. They differ in their understanding of AMR and the responsibility associated with it. Working collaboratively within a VHHC situation, potential challenges might occur due to disagreements or a lack of understanding of other people's perspectives. Having different core values regarding the motives for treatment might result in different treatment thresholds among farmers and veterinarians, with potential differences in the decision-making process and preferred solutions. Reasons for this have been discussed in the literature [(7), p. 14–6], and economic models propose that people choose to act based on the maximum expected utility. However, this is not always the case as there is evidence that farmers do not always decide whether to treat an animal based on economic reasoning (17).

It has also been suggested that decision-making is affected by the context, as well as ways of perceiving risk [(47), p. 159–99]. A difference in perception of risk among farmers and veterinarians has been proposed [(15), p. 2–4]. Sorge et al. [(48), p. 1497–9] suggested that this difference may be due to the lack of knowledge among farmers affecting their risk perception, in this case relating to Johne's disease. In the case of AMR, it could be that farmers do not perceive AMR as a risk due to a lack of knowledge of local, global and future consequences. The knowledge deficit model describes how a poor understanding of the scientific reasoning behind any given advice is why lay people may not follow the advice. In other words, the reason for the difference in risk perception and decision-making between the veterinarian (the expert) and the farmer (the lay person) is due to the farmer's lack of knowledge [(49), p. 112]. However, critics of the knowledge deficit model argue that risk assessment is complex and individual, and related to more than just a lack of knowledge [(15), p. 3–6, (49)]. Differences in risk perception due to a lack of knowledge is therefore not likely to be the sole explanation for the differences in attitudes between veterinarians and farmers. Instead, to avoid major disagreements jeopardising the collaboration within VHHC, farmer-specific VHHC have been proposed, where the individual farmer's risk perception and attitudes are explored through dialogue and taken into account [(15), p. 3–6].

In conclusion, the behavioural belief-based factors identified in this section highlight the importance of trying to understand the other party's perspectives and contextual framework, e.g., emotions, individual risk perception and attitudes, to avoid major disagreements that could jeopardise the ability to change AMU behaviour in collaborations between the farmer and the consulting veterinarian.

### Social Norms Affecting the Veterinary Herd Health Consultancy Relationship

Personal beliefs and attitudes contribute to a person's actions related to changing AMU, but these factors are also influenced by other people. The opinions and behaviour of others can influence and modulate a person's response by building “social norms,” which are created as informal guidelines for behaviour within a group. Social norms are enforced through social sanctions, whereby people feel uncomfortable violating norms due to public disapproval possibly causing shame or embarrassment. Alternatively, following social norms can result in reputational benefit and improve one's self-concept [(50), p. 914–25, (51), p. 3–5]. The literature suggests that the relationship with and opinions of other people are important to both farmers and veterinarians in terms of how their social norms are formed over time [(52), p. 2375–9, (32), p. 8–9]. Some social associations are more relevant to veterinarians, some are more relevant to farmers and some are relevant to both. The identified factors related to social norms affecting herd health management and AMU will be summarised according to this division and discussed in the following sections.

#### Social Norms of the Veterinarian

“Social contacts” (Figure 1, intrinsic, common factor) is a factor common to both farmers and veterinarians, but has a different meaning to each group (see below). For veterinarians, colleagues' opinions were identified as particularly important. A lack of support from colleagues over their choice of prescription could lead veterinarians to prescribe against their own judgement [(32), p. 9]. Swiss veterinarians attending peer study groups emphasised the importance of sharing their experience with their peers to gain new knowledge, compare themselves with others and receive new stimuli [(39), p. 12]. It can be argued that veterinarians compare themselves with their peers and follow e.g., practice policies and their colleagues' prescribing choices to stay in line with social norms.

Another point from the figure with specific relevance to the social norms of the veterinarian is “Pressure from surroundings” (Figure 1, intrinsic, veterinarian factor). One aspect of this will be described here, the other in section Social Norms of the Farmer-Veterinarian Interaction as it concerns the farmer–veterinarian relationship directly. As is apparent from the literature, colleagues' opinions are not always perceived positively but rather as a pressure to prescribe in a specific way. A practice policy for AMU was found to be an important factor for prescribing among veterinarians in New Zealand, next after their own training and costs/benefits for the farmer [(30), p. 88]. This may imply that veterinarians do not want to go against or question an existing practice policy in some situations, so they choose to prescribe according to the policy and perhaps against their own judgement. A certain “prescribing etiquette” has been identified within human medicine, i.e., a set of cultural rules defining AMU. These rules are derived from a hospital culture where the autonomous and experience-based prescribing behaviour of senior doctors affects junior doctors. Furthermore, a culture of “non-interference” in colleagues' prescribing choices also exists [(53), p. 190–4]. Another study within human medicine identified certain “rules of the game” for AMU at hospitals. These rules arise due to the prescribing norms and working conditions at hospitals [(54), p. 83–7]. A prescribing etiquette and cultural rules for AMU might also apply to veterinarians. Despite veterinarians working more independently compared with doctors in a hospital setting, AMU choices might still be influenced by colleagues' opinions, as evident from the literature.

#### Social Norms of the Farmer

In relation to farmers, “Social contacts” (Figure 1, intrinsic, common factor) including opinions from a perceived positive reference group—namely other farmers—were identified as important [(52), p. 2375–9]. The concept of being “a good farmer” was introduced in connection with this, meaning the importance of living up to other farmers' perceptions of “good farming” (46, 52). The role of being “a good farmer” encompasses multiple social norms, each of them dictating appropriate behaviour [(55), p. 207–10, (56)]. As identified in the literature, being “a good farmer” can imply achieving high production levels [(46), p. 2729] as well as using extended therapy for mastitis, i.e., treating for more days than recommended by the veterinarian to achieve the best possible treatment outcomes [(52), p. 2374]. Several studies have illustrated that “the good farmer” can have multiple meanings according to the local “rules of the game” [(56, 57), 589–99].

A local understanding of “the good farmer” could be established through communication with other farmers and through opinions from trusted sources, e.g., the veterinarian [(52), p. 2376–7]. In relation to this, it seems relevant to present the concept of “roadside farming.” According to Burton [(55), p. 201–6], “Roadside farming” is characterised by the exchange of social information by farmers. This happens either by presenting their own farm as well as possible by the roadside or by evaluating other farms. Therefore, a local understanding of “the good farmer” might also be established through non-verbal communication, e.g., through “roadside farming.” In conclusion, it is important for farmers to live up to their social contacts' perception of “good farming.” The social norms related to this concept are created through communication (both verbal and non-verbal) with the outside world, and farmers probably choose to live up to the social norms due to an expected reputational benefit.

A second point specific to farmers is “Considerations” (Figure 1, intrinsic, farmer factor). The literature suggests that farmers perceive expectations from the dairy industry regarding rational AMU positively and as something they want to live up to [(58), p. 34–7] [(59), p. 477]. From a normative perspective, this could be explained as an aspect of being “a good farmer.” Furthermore, farmers might be motivated to live up to expectations from the dairy industry if they expect to achieve a reputational benefit from doing so.

#### Social Norms of the Farmer–Veterinarian Interaction

One of the factors related to the farmer–veterinarian relationship is actually specific to veterinarians, but also directly relevant to the interaction between the two (Figure 1, intrinsic, veterinarian factor, “Pressure from surroundings”). The aspect related to colleagues has been described in section Social Norms of the Veterinarian, but the aspect related to the farmer will be described here. The literature suggests that veterinarians experience pressure from their clients, and one of the reasons behind this has been identified as an actual or perceived client demand for antimicrobials [(60), p. 2, (29), p. 44, (43), p. 367, (61), p. 82–3, (42), p. 4]. Another reason for this pressure to prescribe antimicrobials is due to economic considerations for the farmers. Some broad-spectrum antimicrobials are economically attractive to farmers due to the short withdrawal periods, resulting in veterinarians experiencing pressure to prescribe in a less responsible manner—for example the cheapest treatment solution instead of the most suitable product [(32), p. 8, (30), p. 86–7]. Social norms might also explain why veterinarians feel a pressure to prescribe; they may experience social sanctions (e.g., a bad reputation) from the farmer if they refuse to prescribe cheap broad-spectrum antimicrobials. Research within human medicine has shown that local norms for prescribing practices and interpersonal pressure from patients and their relatives, together with the risk of patients relapsing when not treated, influenced AMU at hospitals [(45), p. s. 830–4]. Similar social and cultural influences might be at play in the veterinarian–farmer collaboration, perhaps encouraging the veterinarian to prescribe out of consideration for the continued relationship with the farmer, the risk for the animal or an urge to comply with social norms. There seems to be a disparity between what veterinarians and farmers perceive as the “correct” choice of antimicrobials and the parameters that this choice should be based on. This disparity could cause complications in the VHHC collaboration, and a mutual understanding should therefore be sought and choices related to AMU should preferably be based on scientifically valid general or local evidence (62).

“Scientific knowledge” has been identified as an important guide of both veterinarians' and farmers' behaviour (Figure 1, intrinsic, common factor). However, there is a difference in the perception of “scientific knowledge”: farmers primarily view the veterinarian as a representative of scientific knowledge [(63), p. 147], whereas published literature from veterinary experts is the epitome of “scientific knowledge” for veterinarians themselves [(64), p. 3, (42), p. 4–5, (37), p. 60]. This difference in perception could also affect the veterinarian–farmer collaboration in relation to HHM. Farmers might not appreciate veterinary recommendations based on published literature, as they may find the advice incompatible with the reality on their farm and expect the veterinarian to adjust the advice accordingly [(7), p. 15].

Regarding the “Type of relationship” between the veterinarian and the farmer within a VHHC setting (Figure 1, intrinsic, common factor), the literature suggests that both groups agree on the importance of a good collaboration when working with AMR [(32), p. 11]. A stable school project in Denmark showed that a mutual trust and openness among the participants had a significant influence on the results obtained [(65), p. 2548–50]. A study from the UK also highlighted the importance of established trust between the veterinarian and the farmer in terms of the veterinarian knowing the actual AMU on the farm [(37), p. 58]. A lack of commitment or understanding of the individual farmer's way of farming (e.g., organic farming) was found to negatively influence the relationship from the farmer's perspective [(33), p. 113–4, (66), p. 19–20]. Conversely, veterinarians in France felt that they were stuck in a role as “firefighters” at organic farms and faced difficulties changing this role due to a lack of regular farm visits and farmers' lack of appreciation for advisory services [(67), p. 12–8]. Furthermore, some Flemish veterinarians believed that the farmers' mentality when it came to using antimicrobials led to high AMU, thus discouraging the collaborative effort [(42), p. 2–3]. The influence of farmers' mentality, behaviour, age and knowledge on veterinary prescribing behaviour was mentioned by Swiss veterinarians [(39), p. 8–9]. Furthermore, veterinarians emphasise the importance of regular visits to work preventively to tackle disease instead of focusing on treatments [(35), p. 3–4, (29), p. 42].

The perceived importance of the mutual relationship between both veterinarians and farmers might be explained by the concept of trust [(12), p. 89]. Möllering [(68), p. 4] gave definitions of trust in the following statement: “Trust can be defined, first of all, as a state of favourable expectation regarding other people's actions and intentions. As such it is seen as the basis for individual risk-taking behaviour, co-operation, reduced social complexity, order, social capital, and so on.” Reduced social complexity implies that social interactions can proceed without the constant evaluation of potential actions by those involved [(69), p. 5–35]. By establishing trust within the relationship, veterinarians and farmers can reduce the complexity of their social interaction and need not discuss or evaluate every single outcome of a certain decision.

According to Luhmann [(69), p. 21–6], we are more trusting of a familiar person than a stranger, and establishing a relationship takes time, which may explain why some interviewed veterinarians from Ireland and the Netherlands emphasised the importance of regular herd visits. Luhmann [(69), p. 21–6] also mentions how trust is less likely to be broken within a persistent relationship, such as the relationship between a veterinarian and a farmer, who will most likely have to continue their collaboration over an undefined period of time. When farmers experience a lack of understanding and commitment from their veterinarian, they may also experience a lack of trust. According to Luhmann [(69), p. 21–6], no one wants to take too many risks when initially building up trust within a relationship. This could explain the farmers' mentality negatively affecting the collaboration, as experienced by the Flemish veterinarians surveyed. Another example of these mechanisms can be found in a social science study concerned with VHHC from European countries and the USA, which identified a tendency for veterinarians to prefer farms with intensive farming due to the regular visits and the potential to build up a close relationship with the farmer. In contrast, relationships with farmers from extensive farms were more distant as they had diverging views on the need for consultancy and less regular herd visits, possibly implying a relationship built on less trust, commitment and understanding [(7), p. 15].

In conclusion, both farmers and veterinarians care about other's opinions and these can influence their own opinions and behaviour. Within collaborations such as a VHHC agreement, both parties should be aware of the influence they have on each other. A better understanding of each other's perspectives, wishes and drivers can result in a more purposeful VHHC towards a local and practical rational AMU. In relation to this, building a mutual relationship through dialogue based on trust could reduce social complexity. A theoretical understanding of the mechanisms behind social norms and their impact on individual behaviour is also of importance.

#### Social Norms Shaping Attitudes and Behaviour

Some of the factors placed within the behavioural belief-based factors might also be explained by social norms. Different treatment practices might be a result of social norms developed within the local society of the farmer and the veterinary clinic. The concept of being “a good veterinarian” might be equally as relevant as “the good farmer,” and also shaped by social norms. For example, social norms might explain why veterinarians see it as their duty to alleviate the suffering of animals, since years of education have taught them to do so. It has been proposed that norms are based on beliefs about facts. If new knowledge emerges and changes what is understood as correct, new norms might be created. However, these changes are often delayed due to the difficulties people face when changing norms and admitting the mistakes of former beliefs [(50), p. 931]. The pressure to prescribe experienced by veterinarians might be complicated further due to a potential delay when changing norms that leads to a disparity in beliefs and knowledge on rational AMU among both veterinarians and farmers.

For farmers, the misuse of antimicrobials identified by Raymond et al. [(36), p. 3233–4] and Buller et al. [(37), p. 56] in the previous section on behavioural belief-based factors can also be discussed from a social norm perspective. It has been proposed that some people simply like to violate social norms, also known as “flouting convention,” which could explain the misuse of antimicrobials by farmers. Another perspective on the misuse of antimicrobials might be a disapproval of norms due to reflective judgement [(50), p. 918]. The surveyed farmers in the study by Raymond et al. (36) might be dissatisfied with the legislation related to AMU and want to contribute to a new way of thinking and new social norms. In connection with this, the theory of psychological reactance might also offer an explanation about the farmers' behaviour. If a person's perceived free behaviour is restrained, for example if a farmer is forced to use certain antimicrobials and these must always be prescribed by a veterinarian due to legislation, they may feel motivated to regain their freedom and use the antimicrobials illegally, ignoring the social influence from others (70). It is possible that similar tendencies could be identified for veterinarians, e.g., a delayed response to regulations on the use of critical antimicrobials, we have, however, not found published literature describing such behaviour.

Summarising normative belief-based factors underlines the influence of social norms in the everyday work of veterinarians and farmers—both individually and in their collaboration. In addition, awareness of how social norms can influence and explain attitudes and decisions may help to improve mutual understanding within a VHHC setting.

### Using the Positive Feedback Loop of Self-Efficacy

This section concerns the third of the intrinsic factors, the belief in self-efficacy-based factors. Belief in self-efficacy is a person's trust in their own ability to do something. Without this trust in oneself, it can be difficult to change behaviour. “Experience” seems to be an important aspect in achieving self-efficacy for both farmers and veterinarians (Figure 1, intrinsic, common factor). The literature suggests that for veterinarians, a lack of work experience can affect their trust in their own decisions [(43), p. 367]. Personal experience with specific drugs or treatments also affects veterinarians' decisions [(42), p. 4, (60), p. 2, (30), p. 86, (38), p. 3497–8, (39), p. 11]. Similarly, personal experience also guides the drug choices doctors make at hospitals, where the clinical situation determines the use of antimicrobials independent of formal policy recommendations [(53), p. 193]. The literature suggests similar aspects among farmers, and several studies have identified a large amount of trust in their own treatment experiences [(71), p. 371–2]—sometimes they will trust this even more than the veterinarian's advice [(31), p. 6, (33), p. 113–4, (34), p. 1848–9, (65), p. 2549, (30), p. 86]. Some studies have identified the use of antimicrobials without any input from the veterinarian, which perhaps implies the same thing [(58), p. 33–4, (63), p. 144]. The opposite situation where the veterinarian works as a trusted source of information for the farmer and possibly contributes to an improved belief in self-efficacy has also been identified, as previously mentioned [(30), p. 86, (46), p. 2732] (Figure 1, intrinsic, farmer factor, “Support”).

Besides experience, “Fear” also affects the self-efficacy of both farmers and veterinarians (Figure 1, intrinsic, common factor). The fear of a negative implication on animal welfare if AMU is reduced further was identified for both groups [(37), p. 55–6, (42), p. 4, (32), p. 7, (58), p. 34]. Some farmers also feared a decline in production, as identified in the survey by Jones et al. [(58), p. 34] and the interview study by Golding et al. [(32), p. 7], as well as economic losses in general. Furthermore, the literature suggests that some farmers are scared to change or halt their AMU due to the risk of relapse in their animals [(40), p. 64, (52), p. 2373], indicating that emotions act as a barrier.

Fischer et al. [(46), p. 2731] identified a lack of ability among farm workers to identify sick animals (Figure 1, intrinsic, farmer factor, “Abilities”). The study also identified a sense of apathy among farmers due to external factors present on their farm, e.g., time and economic constraints, sometimes making it difficult to deal with sick animals. A lack of ability and an apathetic attitude can further affect the self-efficacy of farmers.

There is uncertainty surrounding “Motivation” (Figure 1, intrinsic, common factor) for both farmers and veterinarians and how this affects their belief in self-efficacy. For example, the reason for farmers from the UK not wanting to change AMU on their farms after participating in workshops with a focus on the same is unknown [(59), p. 480–3]. However, as identified in a stable school project in Denmark, sharing good examples or solutions increased the motivation for change among farmers, probably because changing one's own practices seems more achievable when others have succeeded in making similar changes [(65), p. 2549]. The importance of seeing a positive effect of measures taken to improve AMU was identified among farmers. Seeing the results of successfully implemented measures increases the motivation to continue, possibly due to a higher level of trust in self-efficacy [(34), p. 1844]. The literature suggests that veterinarians' motivation is influenced by their clients' motivation [(43), p. 368–71, (32), p. 8], e.g., in a positive feedback. Again, there is an element of uncertainty involved—could a lack of motivation for veterinarians be due to a lack of belief in their own ability to affect the farmers' motivation?

According to Bandura [(72), p. 27–32], self-efficacy is an individual's belief that their effort will produce desired effects, affecting their motivation to act. If people truly believe that they have the ability to change something, they are more likely to try to do so. Bandura also highlights the effect of fulfilling valued goals, which results in self-satisfaction and increased motivation. This might explain why farmers' and veterinarians' motivation can be driven by a belief in self-efficacy via positive feedback. The identified fears of a negative impact on animal welfare and a decline in production might be connected to doubts about their ability to act according to their own core values within a restricted AMU setting. By being aware of the different barriers or opportunities for improving an individual's self-efficacy, veterinarians and farmers can better assist each other in increasing the motivation to act.

The following section will describe extrinsic factors that may have an effect on the intention to move towards a more rational AMU, as well as hinder or promote its implementation.

## Extrinsic Factors Affecting the Intention to Move Towards Rational Antimicrobial Use

Extrinsic factors relating to the external framework surrounding the farmer and the veterinarian will be presented in the following sections. The extrinsic factors include three groups: Community & Industry; Culture & Society; Knowledge, Skills & Ability.

### Agricultural Industry and Community Influencing Antimicrobial Use on Farms

Literature suggests that the rural industry (Figure 1, extrinsic, common factor, “Role of industry”) plays an important role in the development of improved AMU for both farmers and veterinarians [(35), p. 3–4, (73), p. 7–8, (74), p. 6, (37), p. 60–1, (46), p. 2732–3]. According to Golding et al. [(32), p. 10], interviewed farmers expressed a need for the industries and the government to lead the development by supporting research, providing specific guidelines and ensuring better prices for farmers' products. However, which partner should take responsibility differs depending on the respondent, with retailers, food companies, national, and international authorities, farm associations, the dairy industry and veterinary organisations all being mentioned [(35), p. 3–4, (32), p. 10, (73), p. 7–8, (37), p. 60–1].

The “Medicine industry” (Figure 1, extrinsic, common factor) represents an important factor in the agriculture industry, and the literature suggests that both farmers and veterinarians are concerned about it in terms of changing AMU. However, ease of administration has primarily been identified as a consideration for veterinarians, whereas farmers are more focused on the price of medicines. For example, surveys completed by veterinarians from Ireland, the Netherlands, Flanders and other European countries indicated that veterinarians consider the ease of administration for both themselves and the farmer when choosing an antimicrobial drug [(64), p. 3, (60), p. 2–3, (42), p. 4], while farmers complain about medicine prices and choose antimicrobial drugs based on withdrawal times [(40), p. 65, (58), p. 33–4, (31), p. 5–6]. In addition, some veterinarians from France requested more knowledge regarding alternative medicines [(40), p. 67], and farmers in a focus group study suggested improved labelling of drugs so that correct dosages, withdrawal times and the appropriate disease indication would appear clearly on the original label [(31), p. 9].

Another aspect of the rural community that both veterinarians and farmers believe influences their intention to change AMU is the “Advisory services” (Figure 1, extrinsic, common factor), which we will discuss in the context of HHM contracts between the two groups. The literature suggests that veterinarians focus on retaining clients, e.g., to ensure income [(29), p. 42, (39), p. 11]. Some veterinarians in the UK do so by making adjustments according to the farmer's economic situation or by compromising their own opinions to avoid conflicts and thereby maintaining client relationships [(32), p. 8–9]. Ohio veterinarians emphasised the importance of advisory services to reduce the need for antimicrobials [(38), p. 3497]. From the farmers' perspective, some have expressed their frustration regarding prices for veterinary assistance and advice [(33), p. 113–4, (36), p. 3237, (31), p. 6, (32), p. 9]. Other farmers believed consultancy was of limited benefit due to different goal setting or perspectives between themselves and the veterinarian [(33), p. 113–4, (65), p. 2549, (34), p. 1848, (67), p. 12–8, (66), p. 19–20]. Some farmers requested more frequent herd health consultancy from their veterinarian [(33), p. 113], and a survey from the UK identified an association between a positive opinion of herd health plans and a high level of knowledge of AMR among farmers [(75), p. 6].

As indicated by the summarised factors of importance relating to “Community & Industry,” veterinarians and farmers are concerned with the same issues, e.g., the role of industry, the medicine industry and veterinary advisory services. However, their perspectives are not always aligned. The collaboration within VHHC can be complicated due to different interests, e.g., intervals between visits. Communication is needed to align expectations for the collaboration and to avoid veterinarians compromising to retain clients. Furthermore, communication could also result in a mutual understanding of what is important to each group, e.g., medicines that are cheaper or easier to administer, or industry- or government-led initiatives to reduce AMR. Not all of these needs should or could be fulfilled within the VHHC collaboration, but working towards a mutual understanding and establishing a common goal within the collaboration could create a sense of unity, which could subsequently promote positive feelings towards the collaboration in general.

The identified factors relating to the role of the industry, the medicine industry and advisory services will vary from country to country. It is relevant to consider different factors depending on the country's history regarding the introduction, development and role of advisory services, the medicinal products available on the national market and the usual role played by the industry. Therefore, the factors must be carefully considered in relation to the context in question.

### Legislation, Consumers, and Culture Influencing Antimicrobial Use on Farms

In terms of “Culture & Society”–based factors, “Legislation” is an important factor for both farmers and veterinarians (Figure 1, extrinsic, common factor). In line with the role of the industry mentioned in the previous section, government initiatives to enforce rational AMU are called for in the international literature. Experts consulted in a study by Carmo et al. [(74), p. 6] agreed that mandatory interventions have a high potential to reduce AMU. Several studies have identified a need for more legislation in the area of AMU [(35), p. 3, (40), p. 69, (46), p. 2732–3, (39), p. 10, (29), p. 44–5, (42), p. 6], but the opposite opinion was also identified in the literature [(63), p. 144–5, (42), p. 6]. Interviewed farmers from the UK expressed concerns about the administrative work and “tick-box” conformity following legislative initiatives [(37), p. 61]. In addition, some of the interviewed farmers felt that legislative restrictions on AMU challenged their economic situation and disrupted their business [(32), p. 9–10]. Swiss farmers and veterinarians stated that no penalty should be given to farmers with high AMU [(73), p. 7]. The different attitudes towards legislation and the role of the government might depend on the country in which the study was conducted. As illustrated by Postma et al. (42), the surveyed veterinarians from the Netherlands and Flanders had differing opinions on governmental restrictions on AMU. This might be due to the different legislative history of the two countries. At the time of the study, the Netherlands had already experienced legislative restrictions on their AMU and had managed to reduce their AMU without compromising animal welfare, resulting in a more positive attitude towards governmental restrictions. In contrast, Flanders had not yet gone through these changes, possibly explaining their more sceptical attitude towards the possibility of reducing their AMU. A similar tendency was found in the study by Swinkels et al. (52), who found that the interviewed dairy farmers from Germany and the Netherlands also had different opinions on governmental restrictions depending on their country's history and their production structure.

Two farmer-specific points were identified in the literature, namely “Consumers” and “Media and society” (Figure 1, extrinsic, farmer factor). Farmers perceive society as a negative reference group due to a lack of support and understanding of the dairy production process. Interviewed farmers from Sweden, Germany and the Netherlands expressed their frustrations about society due to a simplified and judgemental view of AMU in livestock production and a lack of appreciation of their work in food production in general [(52), p. 2377–8, (46), p. 2732–3]. The media is not perceived as a trusted source of information regarding AMR, and some farmers felt that it assisted in creating a skewed view of agriculture [(32), p. 7, (46), p. 2732–3]. Swedish farmers were also frustrated with the double standards among consumers regarding animal health and environmental issues [(46), p. 2732], and some farmers from Tennessee mentioned a lack of knowledge among consumers, causing misunderstandings about milk marketing [(31), p. 7–9]. However, farmers from the UK also acknowledged the potential for consumers to drive an improvement in AMU by demanding certain product standards [(32), p. 9–10]. To our knowledge, concerns regarding consumers, media and society have not been identified for veterinarians within the literature. This might be due to the less direct effect on their profession, as opposed to the livestock industry, which instantly feels the economic consequences of a downturn in demand.

Within “Culture & Society,” different treatment cultures were also identified in the literature (Figure 1, extrinsic, common factor). The factor “Treatment culture” is defined as treatment options that have been shaped by the respective country. This is exemplified by the questionnaire study by Espetvedt et al. [(61), p. 86], where Norwegian, Swedish, Finnish and Danish veterinarians were asked about their treatment thresholds for mild clinical mastitis. Differences in treatment thresholds across the four countries were identified and reasons behind this hypothesised, e.g., due to differences in pathogens, herd size and farming systems, distance between herds and country geography in general, as well as differences in penalties, herd health programmes and legislation. Treatment culture is not only valid for veterinarians, farmers too are affected by the situation in their specific country. For example, surveyed farmers from Tennessee requested treatment protocols to guide their AMU [(31), p. 10]. Farmers from other areas of the USA also stated a need for protocols, but few actually used them [(36), p. 3231, (71), p. 373]. The lack of treatment protocols or a reluctance to follow them as a part of farming culture could lead to unnecessary use of antimicrobials, thereby creating a country-specific treatment culture.

Looking into the VHHC collaboration, communication between the veterinarian and the farmer is important for achieving a mutual understanding of things that are perceived as important by each side, as seen with the “Community & Industry”–based factors. In terms of “Culture & Society”–based factors, this includes attitudes towards legislative restrictions and—specifically for the farmers—how consumers, media and society are perceived. Again, a mutual understanding and a common goal could create a sense of unity, which could give rise to a positive attitude towards the collaboration in general.

When comparing different countries in relation to AMU and factors of importance for changing AMU, it is important to be aware of the agricultural framework of the countries of interest. As previously stated in this section, treatment cultures seem to be dependent on the country in question, as well as legislation and the role of consumers and society in general. Therefore, it is important to contextualise for national conditions.

### Availability of Resources Influencing Antimicrobial Use on Farms

This section concerns the last of the extrinsic factors, the “Knowledge, Skills & Ability”–based factors. According to the literature, veterinarians and farmers agree on the overall importance of “Knowledge,” “Economics,” “Information and communication,” “Physical framework” and “Diagnostics” when addressing the resources available to support a change in AMU.

In terms of “Knowledge” (Figure 1, extrinsic, common factor), both groups are focused on further education as a key factor in changing AMU [(35), p. 4–5, (74), p. 3–11, (38), p. 3496–7, (64), p. 5, (60), p. 4, (31), p. 7–11, (71), p. 373, (63), p. 144–7]. Several studies have identified a lack of knowledge of AMR among farmers [(31), p. 7, (37), p. 50–2, (75), p. 6, (40), p. 67, (44), p. 1–9], and younger veterinarians have been identified as being more knowledgeable about AMR compared with their older colleagues [(38), p. 3497]. Furthermore, veterinarians focus on the need for research on AMR [(64), p. 5–6, (35), p. 6].

Besides a lack of knowledge, the economic situation of the veterinary practice can influence the intention to change AMU for veterinarians (Figure 1, extrinsic, common factor, “Economics”), but this depends on the country-specific legislation and economic structure relevant to the veterinary practice. Veterinarians across all Nordic countries are only allowed to profit marginally from the sale of antimicrobials (61). If a larger proportion of veterinary income could be derived from the sale of antimicrobials, this may lead to more frequent prescribing [(60), p. 2–4]. There was an association between years of work experience and an expressed need to retain the right to sell and earn money on antimicrobials among Dutch veterinarians [(43), p. 367]. However, in another Dutch study, interviewed veterinarians declared that pharmacy incomes did not drive antimicrobial prescription [(29), p. 45]. In France, the veterinary profession has been accused of contributing to the increasing AMR due to their professional conflict of interest as medicine sales make up a large proportion of their income. This led to them redefining the veterinary position in the public debate on AMR [(76), p. 3–7]. Another aspect of “Economics” is the farmer's economic situation, which is often regarded as a limitation to changing AMU by both veterinarians and farmers [(32), p. 8, (71), p. 373, (35), p. 3, (46), p. 2731, (43), p. 368–71, (29), (42), p. 2].

In line with the economic situation, the “Physical framework” of the farm often challenges change (Figure 1, extrinsic, common factor). The importance of good management [(31), p. 7, (34), p. 1844–8, (59), p. 481–2, (37), p. 57], climate and housing conditions [(43), p. 370, (42), p. 2–3, (32), p. 11], quality of feed [(43), p. 368, (42), p. 3] and biosecurity [(35), p. 5, (36), p. 3234, (42), p. 2–3, (74), p. 6–7] are all emphasised by both farmers and veterinarians. An ethnographic study conducted in East Africa concluded that antimicrobials often became a “quick fix” for a lack of hygiene among citizens [(28), p. 3–4]. A similar tendency for antimicrobial misuse could be a consequence of poor hygiene at dairy farms. The literature suggests an apathetic attitude among farmers and veterinarians towards the physical framework at farms and the challenges this causes. This could imply a shifting focus from changing individual behaviour to an institutional focus as a prerequisite for change. Instead of farmers taking responsibility by renovating and improving their farm facilities and management, conditions for farming in general could be improved at a national level. Continuing to describe the factor “Physical framework,” time constraints faced by farmers could challenge changes in AMU for both the farmers themselves and their affiliated veterinarian [(43), p. 368, (32), p. 9, (71), p. 373, (46), p. 2731, (40), p. 64]. Furthermore, some veterinarians agree on the importance of reliable and accurate farm data on AMU and herd performance in evaluating farm-specific AMU and identifying areas for improvement [(29), p. 42, (35), p. 4, (74), p. 11]; however, we did not identify the same focus from farmers within the included literature.

Literature suggests a mutual focus on the importance of communication skills when addressing AMU and AMR (Figure 1, extrinsic, common factor, “Information and communication”). A lack of communication skills [(32), p. 14] and communication on the topic in general was highlighted by both veterinarians and farmers [(71), p. 370, (60), p. 4, (38), p. 3496]. Relevant stakeholders in Ireland have requested more information on AMR and for this to be communicated in an effective way [(35), p. 5].

Lastly, “Diagnostics” (Figure 1, extrinsic, common factor), including availability, prices and usefulness, leads to frustrations among both veterinarians and farmers. Interviewed farmers from New Zealand were not convinced of the usefulness of bacterial culture since their veterinarian's prescriptions were not affected by the results [(30), p. 86]. Several studies identified limitations in the diagnostics available, e.g., due to costs, sampling difficulties, the time required, the variable and multiple pathogenic results, and the veterinarians' own experience conflicting with the results [(29), p. 43, (64), p. 3, (32), p. 8, (74), p. 4, (44), p. 6]. However, the literature suggests that both veterinarians and farmers agree that valid diagnostics are important and should be implemented further [(35), p. 4–5, (31), p. 9, (36), p. 3236, (64), p. 4, (32), p. 8, (74), p. 8–9].

Several “Knowledge, Skills & Ability”–based factors are therefore important when looking at the resources required to assist a change in AMU according to veterinarians and farmers, and both groups seem to be concerned about the same factors. However, communication remains important as the individual farmer or veterinarian might have different needs [(77), p. 1303–4]. One could imagine a newly educated veterinarian being employed as the herd consultant at a farm with no history of using diagnostics in mastitis treatment. In this case, the veterinarian might not need knowledge of AMR. However, the veterinarian might perceive that the farmer lacks knowledge about both mastitis diagnostics and AMR. Only through communication and by striving to understand each other's perspectives can they agree on a plan that both parties accept.

As with the other identified factors, not all the “Knowledge, Skills & Ability”–based factors are of equal relevance across all countries. It is possible to imagine that there are different traditions in the use of diagnostics, physical frameworks of farming and the level of knowledge about AMR across different countries. Therefore, it is important to contextualise according to national conditions.

## Changes in Antimicrobial Use From Individual vs. Societal Perspectives and Future Prospects

This review of relevant factors in the journey towards rational AMU in dairy cattle herds within a VHHC setting has shown that veterinarians and farmers emphasise more national-oriented solutions as well as those related to the local collaboration. Examples include the request for support from the dairy industry and sector organisations, as well as a revised VHHC framework. In addition, there was a call for balanced legislation on AMU that will not compromise animal welfare or herd finances, and a new discourse on AMU in media and among consumers. These are all examples of areas in which national or structural solutions are demanded by farmers and/or veterinarians.

As mentioned in the introduction to the methodology used in this article (section Overview of Factors Affecting the Intention to Move Towards Rational Antimicrobial Use: A Socio-Ecological Model), the focus on individual behavioural change as a way to reduce AMU, as embedded in the Theory of Planned Behaviour, has been criticised. Instead, there is an emphasis on the need to understand the structural dimensions related to AMU. However, the literature on which this review is based has illustrated that farmers and veterinarians call for both approaches. Due to the type of research, e.g., interview studies that take the individual farmer or veterinarian and their perspectives as a starting point, much of the included literature tends to focus on conclusions at the individual level. These individual solutions will be relevant in an everyday situation, as well as being the continual focus of the local VHHC. However, the factors mentioned by farmers and veterinarians in the included literature, which lie beyond the framework for individual action and in their opinion call for national and international solutions, underlines the need to elaborate the farmer–veterinarian collaboration and include and understand the relevant context. To study these elements, there is a need for a change in research methodology.

Researchers within the field of social sciences have used other methodological approaches to understand the field or the context surrounding AMU. They often take a societal starting point as opposed to an individual one by mapping e.g., the discourse (25), actors and stakeholders (76), social and biological processes (78), infrastructure (28) and networks (54) relevant to AMU.

The approach in this article is reminiscent of a societal approach. We used a model that originally built on the Theory of Planned Behaviour as a structural framework to map all the relevant factors for farmers and veterinarians, and to outline the differences and potential challenges these differences can cause in the VHHC collaboration. However, it is clear from social science research within the area that the context includes more than just national differences in e.g., legislation, the economic model and daily tasks of veterinary practices, available diagnostics and medicines. It is also about discourse and connections between historical, economical and farming structure developments and social and biological processes (25, 27, 78, 79). These structures and developments all become entangled in the individual veterinarian's or farmer's lifeworld, as well as in their mutual collaboration.

The literature that met the inclusion criteria of this review was primarily conducted within the veterinary research area. It investigates farmers' and veterinarians' perception of AMU and their possibility to change it within the VHHC setting. Analysis of the literature has clarified that there is more at play in the farmer–veterinarian collaboration than just economic and rational considerations. Social and cultural norms in the form of specific “rules of the game,” a ritualised AMU, different perceptions of risk, a “prescribing etiquette,” “the good farmer,” “the good veterinarian,” “treatment culture” and emotions such as frustration and fear could potentially shape the collaboration and the possibility to change AMU. The modified Theory of Planned Behaviour used in this article has not directly exposed nor explained any of these mechanisms, rather it has thematised the factors of importance. These factors have been explained and elaborated further through theoretical concepts to better understand the context surrounding the farmer–veterinarian collaboration when working with AMU.

As a result, there is a need for more studies with a focus on both individual actions and the structures surrounding them. The individual actions are those relevant to the daily life of a veterinarian and a farmer working together and making individual and collaborative decisions on AMU. However, the structures surrounding them are just as important as they permeate and affect their local realities. We have limited knowledge on the effect of changes in social and biological processes on farmers' and veterinarians' motivation and AMU levels over time. Therefore, studies conducted in the intersection of qualitative and quantitative research to investigate the actual level of AMU and the motivation to change this over time within the HHM setting are needed.

Furthermore, there is a need to combine the methodological approaches of veterinary and social science literature. A more holistic approach, intertwining the theoretical perspectives of the two research areas, will work synergistically to address the required change in AMU in dairy cattle. The research should acknowledge the fact that everyday decisions and actions related to AMU lie in the collaboration between farmers and veterinarians. However, this must be combined with reflections on the effect of the outside world, which surrounds and defines the farmers' and veterinarians' local mode of action.

## Conclusion

We have summarised the available international literature on factors that influence farmers' and veterinarians' intention to use antimicrobials rationally. This has made it easier to interpret this knowledge in relation to VHHC, which comprises one of the primary settings for working with rational AMU in the production animal sector. Awareness of the identified factors within VHHC can improve the effort to reduce AMU. New perspectives have nuanced the understanding of why and how many of the identified factors are at play within this collaborative context. Important topics have been identified, such as social norms including pressure from social networks, diverging risk perceptions and the importance of trust in the working collaboration. This highlights the importance of communication in improving the understanding of other people's perspectives as well as common goal setting within VHHC. We have identified that not all factors are of equal interest across countries, e.g., legislation and types of advisory services. Moreover, the economic models for veterinary practices differ from country to country, affecting the specific meaning and importance of a given factor.

The included literature and research, which was conducted primarily within the field of veterinary research, focuses on the individual farmer and/or veterinarian and their perspectives on AMU and potential for change within the VHHC setting. However, the review study has identified a request from both farmers and veterinarians for national or international solutions to the AMR problem, for example support from the industry and a new discourse among consumers and media. These solutions go beyond an individual's frame of action. Within the field of social sciences, there has been a focus on the structural dimensions related to AMU, supporting the need for and investigating these national and international perspectives. We argue that future research would benefit from a combined focus on the individual and collaborative actions of farmers and veterinarians within the VHHC setting that frames the everyday choices of AMU in intensive dairy farming. However, the overall structural framework (historical, biological, economical, etc.) surrounding and defining the actions of farmers and veterinarians must also be considered. We have therefore identified a need for studies that bridge the theoretical perspectives of veterinary research and social sciences to understand the potential to change AMU within VHHC in dairy cattle farming.

## Author Contributions

NS, DL, and LN contributed to the design and conception of the review study. The literature search was conducted by NS. Analysis and discussions of the included literature were performed by NS, DL, CJ, and LN. NS produced the first draft of the article, which was redrafted, and edited by DL, CJ, and LN. All authors contributed to the article and approved the submitted version.

## Conflict of Interest

The authors declare that the research was conducted in the absence of any commercial or financial relationships that could be construed as a potential conflict of interest.

## References

[1] Tang KL Caffrey NP Nóbrega DB Cork SC Ronksley PE Barkema HW. Restricting the use of antibiotics in food-producing animals and its associations with antibiotic resistance in food-producing animals and human beings: a systematic review and meta-analysis. Lancet Planet Heal. (2017) 1:e316–27. 10.1016/S2542-5196(17)30141-9 29387833 PMC5785333

[2] Laxminarayan R Duse A Wattal C Zaidi AKM Wertheim HFL Sumpradit N. Antibiotic resistance—the need for global solutions. Lancet Infect Dis. (2013) 13:1057–98. 10.1016/S1473-3099(13)70318-9 24252483

[3] European Commission Guidelines for the Prudent Use of Antimicrobials in Veterinary Medicine. Official Journal of the European Union, C 299, 11.09.2015. (2015) Available online at: https://ec.europa.eu/health/sites/health/files/antimicrobial_resistance/docs/2015_prudent_use_guidelines_en.pdf (accessed January 31, 2020).

[4] Krömker V Leimbach S. Mastitis treatment - reduction in antibiotic usage in dairy cows. Reprod Domest Anim. (2017) 52(Suppl. 3):21–9. 10.1111/rda.13032 28815847

[5] Carmo LP Bouzalas I Nielsen LR Alban L Martins da Costa P Müntener C. Expert opinion on livestock antimicrobial usage indications and patterns in Denmark, Portugal and Switzerland. Vet Rec Open. (2018) 5:1–10. 10.1136/vetreco-2018-000288 30245833 PMC6144899

[6] Woods A Is prevention better than cure? The rise and fall of veterinary preventive medicine, c.1950-1980. Soc Hist Med. (2013) 26:113–31. 10.1093/shm/hks031

[7] Bonnaud L Fortané N Being a vet: the veterinary profession in social science research. Rev Agric Food Environ Stud. (2020) 1–25. 10.1007/s41130-020-00103-1

[8] LeBlanc SJ Lissemore KD Kelton DF Duffield TF Leslie KE. Major advances in disease prevention in dairy cattle. J Dairy Sci. (2006) 89:1267–79. 10.3168/jds.S0022-0302(06)72195-6 16537959

[9] Nir O. What are production diseases, and how do we manage them? Acta Vet Scand Suppl. (2003) 98:21–32. 10.1186/1751-0147-44-S1-S21 15259777

[10] Enevoldsen C Det Israelske Rådgivningskoncept - Og En Dansk Oversættelse. Middelfart: Danske Kvægfagdyrlægers Årsmøde (1997).

[11] da Silva JC Noordhuizen JPTM Vagneur M Bexiga R Gelfert CC Baumgartner W. Veterinary dairy herd health management in Europe constraints and perspectives. Vet. (2006) 28:23–32. 10.1080/01652176.2006.9695203 16605158

[12] Ruston A Shortall O Green M Brennan M Wapenaar W Kaler J. Challenges facing the farm animal veterinary profession in england: a qualitative study of veterinarians' perceptions and responses. Prev Vet Med. (2016) 127:84–93. 10.1016/j.prevetmed.2016.03.008 27094145

[13] Fortané N Antimicrobial resistance: preventive approaches to the rescue? Professional expertise and business model of French Industrial veterinarians. Rev Agric Food Environ Stud. (2020) 1–26. 10.1007/s41130-019-00098-4 PMC7149086 38624270

[14] Andersen HJ Enevoldsen C Towards a Better Understanding of the Farmer' s Management Practices – the Power of Combining Qualitative and Quantitative Data (Chapter in Dissertation). Department of Production Animals and Horses, The Royal Veterinary and Agricultural University, Copenhagen, Denmark (2004).

[15] Kristensen E Jakobsen EB. Challenging the myth of the irrational dairy farmer; understanding decision-making related to herd health. N Z Vet. (2011) 59:1–7. 10.1080/00480169.2011.547162 21328151

[16] Lam T Jansen J van den Borne B Renes R Hogeveen H. What veterinarians need to know about communication to optimise their role as advisors on udder health in dairy herds. N Z Vet. (2011) 59:8–15. 10.1080/00480169.2011.547163 21328152

[17] Garforth C. Livestock keepers' reasons for doing and not doing things which governments, vets and scientists would like them to do. Zoonoses Public Health. (2015) 62:29–38. 10.1111/zph.12189 25903493

[18] Bokma J Dewulf J Deprez P Pardon B Risk factors for antimicrobial use in food-producing animals: disease prevention and socio-economic factors as the main drivers? Vlaams Diergeneeskd Tijdschr. (2018) 87:188–200. 10.21825/vdt.v87i4.16066

[19] Wu Z Antimicrobial use in food animal production: situation analysis and contributing factors. Front Agric Sci Eng. (2018) 5:301–11. 10.15302/J-FASE-2018207

[20] Ritter C Jansen J Roche S Kelton DF Adams CL Orsel K. Determinants of farmers' adoption of management-based strategies for infectious disease prevention and control. J Dairy Sci. (2017) 100:3329–47. 10.3168/jds.2016-11977 28237585

[21] Murphy D Ricci A Auce Z Beechinor J Gabriel BH. EMA and EFSA joint scientific opinion on measures to reduce the need to use antimicrobial agents in animal husbandry in the European Union, And the Resulting Impacts on Food Safety (RONAFA). EFSA. (2017) 15:1–245. 10.2903/j.efsa.2017.4666 32625259 PMC7010070

[22] Ellis-Iversen J Cook AJC Watson E Nielen M Larkin L Wooldridge M. Perceptions, circumstances and motivators that influence implementation of zoonotic control programs on cattle farms. Prev Vet Med. (2010) 93:276–85. 10.1016/j.prevetmed.2009.11.005 19963291

[23] Panter-Brick C Clarke SE Lomas H Pinder M Lindsay SW. Culturally compelling strategies for behaviour change: a social ecology model and case study in malaria prevention. Soc Sci Med. (2006) 62:2810–25. 10.1016/j.socscimed.2005.10.009 16352385

[24] Ajzen I. The theory of planned behavior. Organ Behav Hum Decis Process. (1991) 50:179–211. 10.1016/0749-5978(91)90020-T21929476

[25] Chandler CIR. Current accounts of antimicrobial resistance: stabilisation, individualisation and antibiotics as infrastructure. Palgrave Commun. (2019) 5:1–13. 10.1057/s41599-019-0263-4 31157116 PMC6542671

[26] Rynkiewich K Finding What's Wrong With Us: antibiotic prescribing practice among physicians in the United States. Front Sociol. (2020) 5:1–9. 10.3389/fsoc.2020.00005 PMC8022448 33869414

[27] Broom A Kenny K Prainsack B Broom J Antimicrobial Resistance as a Problem of Values? Views from Three Continents. Crit Public Health. (2020) 1–13. 10.1080/09581596.2020.1725444

[28] Denyer Willis L Chandler C. Quick fix for care, productivity, hygiene and inequality: reframing the entrenched problem of antibiotic overuse. BMJ Glob Heal. (2019) 4:1–6. 10.1136/bmjgh-2019-001590 31497315 PMC6703303

[29] Speksnijder DC Jaarsma ADC van der Gugten AC Verheij TJM Wagenaar JA. Determinants associated with veterinary antimicrobial prescribing in farm animals in the Netherlands: a qualitative study. Zoonoses Public Health. (2015) 62:39–51. 10.1111/zph.12168 25421456

[30] McDougall S Compton CWR Botha N. Factors influencing antimicrobial prescribing by veterinarians and usage by dairy farmers in New Zealand. N Z Vet. (2017) 65:84–92. 10.1080/00480169.2016.1246214 27748166

[31] Ekakoro JE Caldwell M Strand EB Okafor CC. Drivers of antimicrobial use practices among tennessee dairy cattle producers. Vet Med Int. (2018) 2018:1–14. 10.1155/2018/1836836 30687493 PMC6327273

[32] Golding SE Ogden J Higgins HM. Shared goals, different barriers: a qualitative study of uk veterinarians' and farmers' beliefs about antimicrobial resistance and stewardship. Front Vet Sci. (2019) 6:1–17. 10.3389/fvets.2019.00132 31106216 PMC6494936

[33] Vaarst M Thamsborg SM Bennedsgaard TW Houe H Enevoldsen C Aarestrup FM Organic dairy farmers' decision making in the first 2 years after conversion in relation to mastitis treatments. Livest Prod Sci. (2003) 80:109–20. 10.1016/S0301-6226(02)00310-X

[34] Vaarst M Bennedsgaard TW Klaas I Nissen TB Thamsborg SM Østergaard S. Development and daily management of an explicit strategy of nonuse of antimicrobial drugs in twelve danish organic dairy herds. J Dairy Sci. (2006) 89:1842–53. 10.3168/jds.S0022-0302(06)72253-6 16606756

[35] Magalhães-Sant Ana M More SJ Morton DB Hanlon AJ. Challenges facing the veterinary profession in Ireland: 2. On-Farm Use of Veterinary Antimicrobials. Ir Vet. (2017) 70:28:1–9. 10.1186/s13620-017-0106-9 28932389 PMC5602862

[36] Raymond MJ Wohrle RD Call DR. Assessment and promotion of judicious antibiotic use on dairy farms in washington state. J Dairy Sci. (2006) 89:3228–40. 10.3168/jds.S0022-0302(06)72598-X 16840641

[37] Buller H Hinchliffe S Hockenhull J Barrett D Reyher K Butterworth A Systematic Review and Social Research to Further Understanding of Current Practice in the Context of Using Antimicrobials in Livestock Farming and to Inform Appropriate Interventions to Reduce Antimicrobial Resistance Within the Livestock Sector. London (2015) Available online at: http://randd.defra.gov.uk/Default.aspx?Menu=Menu&Module=More&Location=None&Completed=0&ProjectID=19623 (accessed February 05, 2020).

[38] Cattaneo AA Wilson R Doohan D LeJeune JT. Bovine veterinarians' knowledge, beliefs, and practices regarding antibiotic resistance on ohio dairy farms. J Dairy Sci. (2009) 92:3494–02. 10.3168/jds.2008-1575 19528628

[39] Pucken VB Schü Pbach-Regula G Gerber M Gross CS Bodmer M Loor JJ. Veterinary peer study groups as a method of continuous education-a new approach to identify and address factors associated with antimicrobial prescribing. PLoS ONE. (2019) 14:e0222497. 10.1371/journal.pone.0222497 31536527 PMC6752762

[40] Poizat A Bonnet-Beaugrand F Rault A Fourichon C Bareille N. Antibiotic use by farmers to control mastitis as influenced by health advice and dairy farming systems. Prev Vet Med. (2017) 146:61–72. 10.1016/j.prevetmed.2017.07.016 28992929

[41] Broom A Kirby E Gibson AF Post JJ Broom J. Myth, manners, and medical ritual: defensive medicine and the fetish of antibiotics. Qual Health Res. (2017) 27:1994–2005. 10.1177/1049732317721478 28737082

[42] Postma M Speksnijder DC Jaarsma ADC Verheij TJM Wagenaar JA Dewulf J. Opinions of veterinarians on antimicrobial use in farm animals in flanders and the Netherlands. Vet Rec. (2016) 16:1–10. 10.1136/vr.103618 27313178

[43] Speksnijder DC Jaarsma DAC Verheij TJM Wagenaar JA. Attitudes and perceptions of Dutch veterinarians on their role in the reduction of antimicrobial use in farm animals. Prev Vet Med. (2015) 121:365–73. 10.1016/j.prevetmed.2015.08.014 26341466

[44] Helliwell R Morris C Raman S Can resistant infections be perceptible in UK dairy farming? Palgrave Commun. (2019) 5:1–9. 10.1057/s41599-019-0220-2

[45] Broom A Broom J Kirby E Adams J The social dynamics of antibiotic use in an Australian hospital. J Sociol. (2016) 52:824–39. 10.1177/1440783315594486

[46] Fischer K Sjöström K Stiernström A Emanuelson U. Dairy farmers' perspectives on antibiotic use: a qualitative study. J Dairy Sci. (2019) 102:2724–37. 10.3168/jds.2018-15015 30612802

[47] Pindyck RS Rubinfeld DL Microeconomics. 8th ed Upper Saddle River, NJ: Prentice Hall (2013).

[48] Sorge U Kelton D Lissemore K Godkin A Hendrick S Wells S Attitudes of Canadian dairy farmers toward a voluntary johne's disease control program. J Dairy Sci. (2010) 93:1491–9. 10.3168/jds.2009-2447 20338426

[49] Hansen J Holm L Frewer L Robinson P Sandøe P Beyond the knowledge deficit: recent research into lay and expert attitudes to food risks. Appetite. (2003) 41:111–21. 10.1016/S0195-6663(03)00079-5 14550309

[50] Sunstein CR Social norms and social roles. Columbia Law Rev. (1996) 96:903 10.2307/1123430

[51] Coent P Le PR Thoyer S Do Farmers Follow the Herd? The Influence of Social Norms in the Participation to Agri-Environmental Schemes. Montpellier: Center for Environmental Economics (2018).

[52] Swinkels JM Hilkens A Zoche-Golob V Krömker V Buddiger M Jansen J. Social Influences on the duration of antibiotic treatment of clinical mastitis in dairy cows. J Dairy Sci. (2015) 98:2369–80. 10.3168/jds.2014-8488 25682148

[53] Charani E Castro-Sanchez E Sevdalis N Kyratsis Y Drumright L Shah N. Understanding the determinants of antimicrobial prescribing within hospitals: the role of Prescribing Etiquette. *Clin Infect Dis*. (2013) 57:188–96. 10.1093/cid/cit212 23572483 PMC3689346

[54] Broom A Broom J Kirby E. Cultures of resistance? A Bourdieusian Analysis of Doctors' Antibiotic Prescribing. Soc Sci Med. (2014) 110:81–8. 10.1016/j.socscimed.2014.03.030 24727665

[55] Burton RJF Seeing through the Good Farmer's eyes: towards developing an understanding of the social symbolic value of Productivist behaviour. Sociol Ruralis. (2004) 44:195–215. 10.1111/j.1467-9523.2004.00270.x

[56] Sutherland L-A Darnhofer I Of organic farmers and Good Farmers: changing habitus in rural England. J Rural Stud. (2012) 28:232–40. 10.1016/j.jrurstud.2012.03.003

[57] Shortall O Sutherland LA Ruston A Kaler J True cowmen and commercial farmers: exploring vets' and dairy farmers' contrasting views of Good Farming in relation to biosecurity. Sociol Ruralis. (2018) 58:583–603. 10.1111/soru.12205

[58] Jones PJ Marier EA Tranter RB Wu G Watson E Teale CJ. Factors affecting dairy farmers' attitudes towards antimicrobial medicine usage in cattle in England and wales. Prev Vet Med. (2015) 121:30–40. 10.1016/j.prevetmed.2015.05.010 26123631

[59] van Dijk L Hayton A Main DCJ Booth A King A Barrett DC. Participatory policy making by dairy producers to reduce anti-microbial use on farms. Zoonoses Public Health. (2017) 64:476–84. 10.1111/zph.12329 28026910

[60] Gibbons JF Boland F Buckley JF Butler F Egan J Fanning S Influences on antimicrobial prescribing behaviour of veterinary practitioners in cattle practice in Ireland. Vet Rec. (2013) 5:1–5. 10.1136/vr.100782 23293148

[61] Espetvedt MN Rintakoski S Wolff C Lind AK Lindberg A Virtala AMK. Nordic veterinarians' threshold for medical treatment of dairy cows, influence on disease recording and medicine use: mild clinical mastitis as an example. Prev Vet Med. (2013) 112:76–89. 10.1016/j.prevetmed.2013.07.004 23948145

[62] Lastein DB Herd-Specific Randomized Trials: An Approach for Effect Evaluation in a Dairy Herd Health Management Program (Dissertation). Department of Large Animal Sciences, University of Copenhagen, Copenhagen, Denmark (2012).

[63] Kramer T Jansen LE Lipman LJA Smit LAM Heederik DJJ Dorado-García A. Farmers' knowledge and expectations of antimicrobial use and resistance are strongly related to usage in Dutch livestock sectors. Prev Vet Med. (2017) 147:142–8. 10.1016/j.prevetmed.2017.08.023 29254712

[64] De Briyne N Atkinson J Pokludová L Borriello SP Price S Factors influencing antibiotic prescribing habits and use of sensitivity testing amongst veterinarians in Europe. Vet Rec. (2013) 1:1–7. 10.1136/vr.101454 PMC3841786 24068699

[65] Vaarst M Nissen TB Østergaard S Klaas IC Bennedsgaard TW Christensen J. Danish stable schools for experiential common learning in groups of organic dairy farmers. J Dairy Sci. (2007) 90:2543–54. 10.3168/jds.2006-607 17430959

[66] Duval JE Bareille N Fourichon C Madouasse A Vaarst M. How can veterinarians be interesting partners for organic dairy farmers? French Farmers' Point of Views. Prev Vet Med. (2017) 146:16–26. 10.1016/j.prevetmed.2017.07.013 28992922

[67] Duval JE Bareille N Fourichon C Madouasse A Vaarst M. Perceptions of French private veterinary practitioners' on their role in organic dairy dairy farmers. Prev Vet Med. (2016) 133:10–21. 10.1016/j.prevetmed.2016.09.008 27720023

[68] Möllering G The nature of trust: from georg simmel to a theory of expectation, interpretation and suspension. Sociology. (2001) 35:403–20. 10.1017/S0038038501000190

[69] Luhmann N Trust and Power. Cambridge: Polity Press (2017).

[70] Steindl C Jonas E Sittenthaler S Traut-Mattausch E Greenberg J. Understanding psychological reactance. Z Psychol. (2015) 223:205–14. 10.1027/2151-2604/a000222 27453805 PMC4675534

[71] Friedman DB Kanwat CP Headrick ML Patterson NJ Neely JC Smith LU. Importance of prudent antibiotic use on dairy farms in south carolina: a pilot project on farmers' knowledge, attitudes and practices. Zoonoses Public Health. (2007) 54:366–75. 10.1111/j.1863-2378.2007.01077.x 18035975

[72] Bandura A. Social cognitive theory: an agentic perspective. Asian J Soc Psychol. (1999) 2:21–41. 10.1111/1467-839X.00024 11148297

[73] van den Borne BHP van Soest FJS Reist M Hogeveen H. Quantifying preferences of farmers and veterinarians for national animal health programs: the example of bovine mastitis and antimicrobial usage in Switzerland. Front Vet Sci. (2017) 4:1–13. 10.3389/fvets.2017.00082 28626750 PMC5454046

[74] Carmo LP Nielsen LR Alban L da Costa PM Schüpbach-Regula G Magouras I. Veterinary expert opinion on potential drivers and opportunities for changing antimicrobial usage practices in livestock in Denmark, Portugal, and Switzerland. Front Vet Sci. (2018) 5:1–14. 10.3389/fvets.2018.00029 29546044 PMC5837977

[75] Higham LE Deakin A Tivey E Porteus V Ridgway S Rayner AC. A Survey of dairy cow farmers in the United Kingdom: knowledge, attitudes and practices surrounding antimicrobial use and resistance. Vet Rec. (2018) 183:1–9. 10.1136/vr.104986 30413678

[76] Fortané N Veterinarian Responsibility: conflicts of definition and appropriation surrounding the public problem of antimicrobial resistance in France. Palgrave Commun. (2019) 5:1–12. 10.1057/s41599-019-0273-2

[77] Jansen J Steuten CDM Renes RJ Aarts N Lam TJGM. Debunking the myth of the hard-to-reach farmer: effective communication on udder health. J Dairy Sci. (2010) 93:1296–306. 10.3168/jds.2009-2794 20172249

[78] Hinchliffe S Butcher A Rahman MM The AMR problem: demanding economies, biological margins, and co-producing alternative strategies. Palgrave Commun. (2018) 4:1–12. 10.1057/s41599-018-0195-4

[79] Landecker H. Antibiotic resistance and the biology of history. Body Soc. (2016) 22:19–52. 10.1177/1357034X14561341 28458609 PMC5390938

